# Sharpening up tumor microenvironment to enhance the efficacy of immune checkpoint blockade on head and neck cancer using a CpG-oligodeoxynucleotide

**DOI:** 10.1007/s00262-021-03062-8

**Published:** 2021-09-28

**Authors:** Jen-Chih Tseng, Jing-Xing Yang, Yi-Ling Liu, Yu-Wen Su, Alan Yueh-Luen Lee, Ya-Wen Chen, Ko-Jiunn Liu, Yunping Luo, Yi-Ren Hong, Tsung-Hsien Chuang

**Affiliations:** 1 Immunology Research Center, National Health Research Institutes, Zhunan, Miaoli 35053 Taiwan; 2 National Institute of Cancer Research, National Health Research Institutes, Zhunan, Miaoli 35053 Taiwan; 3 Department of Immunology, Institute of Basic Medical Sciences, Chinese Academy of Medical Sciences, School of Basic Medicine, Peking Union Medical College, Beijing, 100005 China; 4 Graduate Institute of Medicine, College of Medicine, Kaohsiung Medical University, Kaohsiung, 80708 Taiwan; 5 Program in Environmental and Occupational Medicine, Kaohsiung Medical University, Kaohsiung, 80708 Taiwan

**Keywords:** CpG-oligodeoxynucleotide, Head and neck cancer, Immune checkpoint blockade, Immune stimulator, Toll-like receptor, Tumor microenvironment

## Abstract

Head and neck cancers are a type of life-threatening cancers characterized by an immunosuppressive tumor microenvironment. Only less than 20% of the patients respond to immune checkpoint blockade therapy, indicating the need for a strategy to increase the efficacy of immunotherapy for this type of cancers. Previously, we identified a type B CpG-oligodeoxynucleotide (CpG-ODN) called CpG-2722, which has the universal activity of eliciting an immune response in grouper, mouse, and human cells. In this study, we further characterized and compared its cytokine-inducing profiles with different types of CpG-ODNs. The antitumor effect of CpG-2722 was further investigated alone and in combination with an immune checkpoint inhibitor in a newly developed syngeneic orthotopic head and neck cancer animal model. Along with other inflammatory cytokines, CpG-2722 induces the gene expressions of interleukin-12 and different types of interferons, which are critical for the antitumor response. Both CpG-2722 and anti-programmed death (PD)-1 alone suppressed tumor growth. Their tumor suppression efficacies were further enhanced when CpG-2722 and anti-PD-1 were used in combination. Mechanistically, CpG-2722 shaped a tumor microenvironment that is favorable for the action of anti-PD-1, which included promoting the expression of different cytokines such as IL-12, IFN-*β*, and IFN-*γ*, and increasing the presence of plasmacytoid dendritic cells, M1 macrophages, and CD8 positive *T* cells. Overall, CpG-2722 provided a priming effect for CD8 positive *T* cells by sharpening the tumor microenvironment, whereas anti-PD-1 released the brake for their tumor-killing effect, resulting in an enhanced efficacy of the combined CpG-2722 and anti-PD-1.

**Supplementary Information:**

The online version contains supplementary material available at 10.1007/s00262-021-03062-8.

## Introduction

CpG-oligodeoxynucleotides (CpG-ODNs) are synthetic activators of toll-like receptor 9 (TLR9) and TLR21 in different species. Mammals express TLR9 but lack of TLR21. In contrast, avian species only express TLR21, while fish species contain both TLRs [1, 2]. The activation of mammalian TLR9 by CpG-ODNs induces immune responses including an innate immune response elicited within hours after CpG-ODN stimulation, followed by the second phase of an adaptive immune response that occurs several days later. During this process, the CpG-ODN activated antigen-presenting cells become competent for their antigen presentation and production of Th1 response-promoting cytokines. Increased expression of co-stimulatory molecules enhances the antigen-presenting activity of the cells to naïve *T* cells. The produced cytokines promote a *T* helper (Th) 1 polarized immune response and CD8 positive *T* cells responses with an effective killing activity [3, 4]. Due to the fact that the activated immune response facilitates the eradication of cancer cells from body, the antitumor effect of CpG-ODN was investigated and has been demonstrated in various cancer animal models [5, 6]. In addition, CpG-ODNs are being investigated in clinical trials as a therapeutic agent for cancer treatments, but thus far, no CpG-ODN has yet been approved for cancer therapy [7–9].

The immunostimulatory activity of a CpG-ODN is determined by its nucleotide sequence and structure, which include the content of its CpG-dideoxynucleotides containing hexamer motifs (CpG-hexamer motifs), and the number, position, spacing, and surrounding bases of these CpG-motifs [10, 11]. Based on their structures, CpG-ODNs can be divided mainly into three different immune stimulatory types. Type A CpG-ODNs induce the production of IFN-α and activate the maturation of plasmacytoid dendritic cells (pDCs), but have little effect on *B*-cell activation. Type B CpG-ODNs strongly induce *B*-cell proliferation, cytokine production and have some effect on pDC and monocyte maturation, and NK cell activation. The immune stimulatory property of type C CpG-ODNs is between that of the type A and type B CpG-ODNs [12, 13]. Type B CpG-ODNs are the most commonly used CpG-ODNs. In addition, a CpG-ODN usually has different strengths of activity in different species. This species-specific activity of a CpG-ODN is mainly determined by the nucleotide context of its CpG-hexamer motifs [14–16].

CTLA-4 and PD-1 are the two best investigated immune checkpoint regulators that play important roles in maintaining the homeostasis of the immune system in preventing disorders caused by the over-activation of immune responses. CTLA-4 is essential for immune tolerance and plays a central in the regulation of *T*-cell activation. PD-1 controls the late immune response of *T* cells in peripheral tissues, as its ligands are mainly expressed in non-lymphoid tissues [17–19]. Various CTLA-4 and PD-1/PD-L1 monoclonal antibodies have been developed for anti-tumors by immune checkpoint blockade. An anti-CTLA-4 antibody was approved by the US FDA in 2011; since then, six additional PD-1 or PD-L1 antibodies have been approved for immunotherapy of different cancer types [20, 21]. Cancer therapy with these immune checkpoint inhibitors was demonstrated to have a notable efficacy; nevertheless, the response rate of patients with solid tumors is generally less than 30%. Thus, there is an immense need to improve the efficacy of the therapy with immune checkpoint inhibitors [22, 23].

Head and neck cancers are one of the most common cancers worldwide and head and neck squamous cell carcinoma (HNSCC) accounted for more than 90% of the cancer phenotype. The cancer patients suffer from a poor quality of life. Currently, there is lack of highly effective and satisfied therapeutic strategy for the treatment of this type of cancers. Only less than 20% of the patients respond to therapy with immune checkpoint blockade. This is consistent with the fact that this type of cancer is often characterized by an immunosuppressive microenvironment [24, 25]. Recently, we developed an orthotopic syngeneic mouse model with an immortal cell line derived from mouse HNSCC for studying immunotherapy of the cancers [26]. Further, in previous studies, we developed a CpG-ODN called CpG-2722, which activates human and mouse TLR9s, and fish TLR21 [27]. In this study, we further characterized the immunostimulatory properties of this CpG-2722 and investigated its tumor-suppressive activities alone and in combination with anti-PD-1 using this developed orthotopic syngeneic HNSCC animal model. The CpG-2722 was potent in inducing the expression of IL-12 and IFN-*γ* as type B CpG-ODN, but also induced type I IFNs like type A CpG-ODN. CpG-2722 and anti-PD-1 alone suppressed tumor growth. In addition, a combination of CpG-2722 and anti-PD-1 showed a cooperative effect on the regression of HNSCCs.

## Materials and methods

### Reagents, antibodies, and human peripheral blood mononuclear cells (PBMCs)

All CpG-ODNs were purchased from Integrated DNA Technologies, Inc. CpG-ODNs dissolved in DNase/RNase free water, and aliquots of CpG-ODNs were stored at – 20 ℃. Anti-PD-1 antibody used for *in vivo* treatment was purchased from InvivoGen. (Cat. No. mpd1-mab15-10). Rat anti-mouse CD8 antibody used for immunohistochemistry was purchased from Invitrogen (clone: 4SM15, Cat. No. 14-0808-82). Trizol reagent and SuperScript™ IV kit were purchased from Invitrogen. SYBR® Green PCR kit was purchased from Qiagen. Human PBMCs were purchased from ZenBio, Inc.

### Mouse splenocytes and bone marrow-derived macrophages (BMDMs) preparation

Mouse splenocytes and BMDMs were isolated from 6- to 8-week-old C57BL/6 J mouse (National Laboratory Animal Center, Taiwan). To prepare splenocytes, mouse spleen was collected and pounded by using the plunger of a syringe. Single cells were squeezed out of the spleen fragments, passed through the 40-μm nylon cell strainer (BD FalconTM), and centrifuged at 1500 rpm for 5 min. Cell pellet was resuspended with RBC lysis buffer for 2 min, and the lysis reaction was terminated by adding 30 ml PBS. Splenocytes were spin down and cultured in RPMI 1640 completed medium at 37 °C in a 5% CO_2_ incubator. To prepare BMDMs, bone marrow cells were washed out of tibias and femurs, passed through a 40-μm nylon cell strainer, and centrifuged at 1500 rpm for 5 min. Cell pellet was resuspended with RBC lysis buffer for 2 min, and lysis reaction was terminated by adding 30 ml PBS. Bone marrow cells were spin down and cultured in 70% DMEM completed medium containing 10% FBS, L-glutamine, antibiotics, 10 mM HEPES buffer, and 30% L929 conditional medium at 37 °C in a 5% CO_2_ incubator for 7 days.

### RNA isolation

Total RNA from mouse splenocytes, BMDMs, and human PBMCs was isolated using the illustra™ RNAspin Mini Kit (GE Healthcare) following the manufacturer's protocol. RNA samples from NHRI-HN1-derived tumors were isolated using the TRIzol reagent.

### Reverse transcription-quantitative PCR (RT-qPCR) analysis

Cells were treated with different CpG-ODNs at 0.5 μM for 4 h. RNA samples were then isolated, and reverse transcription was performed using the SuperScript™ IV First-Strand Synthesis System (Invitrogen). We performed quantitative PCR by using QuantiNova™ SYBR® Green PCR Kit (Qiagen) and Applied Biosystems ViiA™ 7 Real-Time PCR System with gene-specific primers (Supplementary Table1 and Supplementary Table 2) for gene expression analysis. The expression level of *β*-actin was used as the loading control.

### Enzyme-linked immunosorbent assay for cytokine production

Human PBMCs were treated with or without different CpG-ODNs as indicated for 24 h, and cell culture media were collected. Cytokines production was measured using enzyme-linked immunosorbent assay (ELISA) kits from eBioscience (San Diego, CA, the USA) following the manufacturer’s protocol.

### Syngeneic orthotopic head and neck cancer animal model

Indicated amount of NHRI-HN1 cells were mixed with matrigel (BD Biosciences) at 1:1 ratio to a total volume of 100 μl. The cells were intramucosally injected into the 6–8-week-old C57BL/6 J mice through a side of buccal region to grow the tumor [28]. When tumors reached the indicated size, the mice were intratumorally injected with the indicated amount of CpG-2722 twice/week, in combination with or without 10 μg of anti-PD-1 antibody once/week. All groups contained five mice and five tumors. Tumor volume of the mice bearing NHRI-HN1-derived tumor was measured using the formula = length × (width)^2^ × 0.5.

### Immunohistochemistry

Paraffin-embedded NHRI-HN1-derived tumors were sectioned into 5-μm tissue slides. These tissue slides were rehydrated with graded concentrations of ethanol to PBS and blocked endogenous peroxidase with 3% hydrogen peroxide for 5 min. For CD8 staining, a rat monoclonal antibody against mouse CD8 (clone: 4SM15, Invitrogen) was used at a dilution of 1:50 and incubated at room temperature for 1 h. The tissue sections were incubated with HRP-conjugated secondary antibody at room temperature for 30 min following washing with PBST. The detection was processed in the Discovery XT automated IHC/ISH slide staining system (Ventana Medical System, Inc. Tucson), using the ultraView Universal DAB Detection Kit (Ventana Medical System, Inc. Tucson), according to the manufacturer’s instructions. Immunostaining was visualized after counterstaining with hematoxylin. CD8-positive cells and leukocyte infiltration were counted using ImageJ software.

## Results

### Induction of cytokine expressions in human cells by CpG-2722

**Fig. 1:**
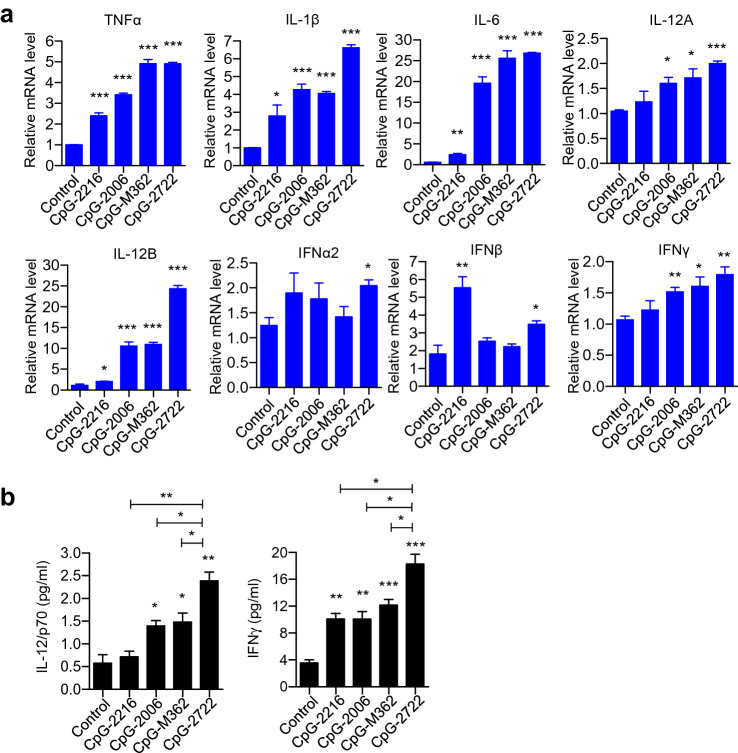
Cytokine-inducing effect of CpG-2722 in human cells. Human peripheral blood mononuclear cells (PBMCs) were treated with 0.5 μM CpG-ODNs as indicated. **a** After 4 h, cells were lysed and relative mRNA levels of different cytokines were determined by RT-qPCR. Expression level of *β*-actin was used as the loading control. **b** After 24 h, cytokines as indicated, secreted into cell culture medium were measured with ELISA. Data represent mean ± SEM (*n* = 3 independent experiments). Asterisk *, **, and *** represent statistically significant difference *p* < 0.05, *p* < 0.01, and < 0.001, respectively, compared to the control

The species-specific activity of a CpG-ODN is determined by its nucleotide sequence and length. For example, CpG-2006 is more potent than CpG-1826 in activating human cells; in contrast, CpG-1826 is more potent than CpG-2006 in activating murine cells [14–16]. As shown in Table 1, the CpG-2006 contains 24 nucleotides and three copies of the GTCGTT-hexamer motif, CpG-1826 contains 20 nucleotides and two copies of the GACGTT-hexamer motif, and CpG-2722 contains 19 nucleotides with two copies of the GTCGTT-hexamer motif and four thymidines between these two hexamer motifs. The CpG-2722 was previously developed for the activation of grouper (*Epinephelus* spp.) TLR21s and also displayed activities on mouse TLR9 and human TLR9; thus, it is an universal CpG-ODN for multiple species [27]. To explore its uses as an immunostimulant in mammals, in this study, we first compared its cytokine induction profiles in parallel with different types of CpG-ODNs. Of them, same as the CpG-2722, CpG-2006 is a type B CpG-ODN containing a phosphorothiolate backbone throughout the entire sequence with three CpG-motifs. CpG-2216 is a type A CpG-ODN with preferential activity for human cells and contains a central phosphodiester palindrome region with a CpG-motif in the palindrome and poly (*G*) sequences and a phosphorothioate backbone attached to the 5′ and 3′ ends. CpG-M362 is a type C CpG-ODN with activities for both of human and mouse cells. This CpG-ODN contains phosphorothioate backbone with one or two CpG-motifs and a palindromic sequence at the 3′ end (Table 1) [12, 13]. Human PBMCs were stimulated with these CpG-ODNs, and expression of different cytokine genes was analyzed with reverse transcription-quantitative polymerase chain reaction (RT-qPCR). The results revealed that CpG-2722 exhibited activities to induce the expression of inflammatory cytokines including TNF-*α*¸IL-1*β*, IL-6, IL-12B, and IFN-*γ* as the CpG-2006 and CpG-M362; nevertheless, it also activated the expression of type I IFNs including IFN*α*2 and IFN-*β* like type A CpG-ODN **(**Fig. 1a). IL-12p70 is a heterodimer of IL-12A and IL-12B. This cytokine and the IFN-*γ* play a key role in promoting* T-*cell proliferation and activation for antitumor responses [29, 30]. Therefore, the production of these two cytokines in the cell culture medium was verified with an ELISA assay. Consistent with its ability of inducing cytokine expressions, the CpG-2722 showed good activities in the induction of IL-12 and IFN-*γ* productions than other CpG-ODNs (Fig. 1b).

**Table 1:** Structural features of CpG-ODNs used in this study. CpG-ODNs in each of the three major types and their species-specific activity are shown. Asterisks stand for phosphorothioate bonds. Otherwise are phosphorodiester bonds

Name	Type	Species-preference	Sequence
CpG-1585	A	Mouse	G*GGGTCAACGTTGAG*G*G*G*G*G
CpG-2216	A	Human	G*GGGGACGATCGTCG*G*G*G*G*G
CpG-1826	B	Mouse	T*C*C*A*T*G*A*C*G*T*T*C*C*T*G*A*C*G*T*T
CpG-2006	B	Human	T*C*G*T*C*G*T*T*T*T*G*T*C*G*T*T*T*T*G*T*C*G*T*T
CpG-2722	B	Mouse/human/fish	G*T*T*G*T*C*G*T*T*T*T*T*T*G*T*C*G*T*T
CpG-M362	C	Mouse/human	T*C*G*T*C*G*T*C*G*T*T*C*G*A*A*C*G*A*C*G*T*T*G*A*T

### Induction of cytokine expressions in mouse cells by CpG-2722

**Fig. 2:**
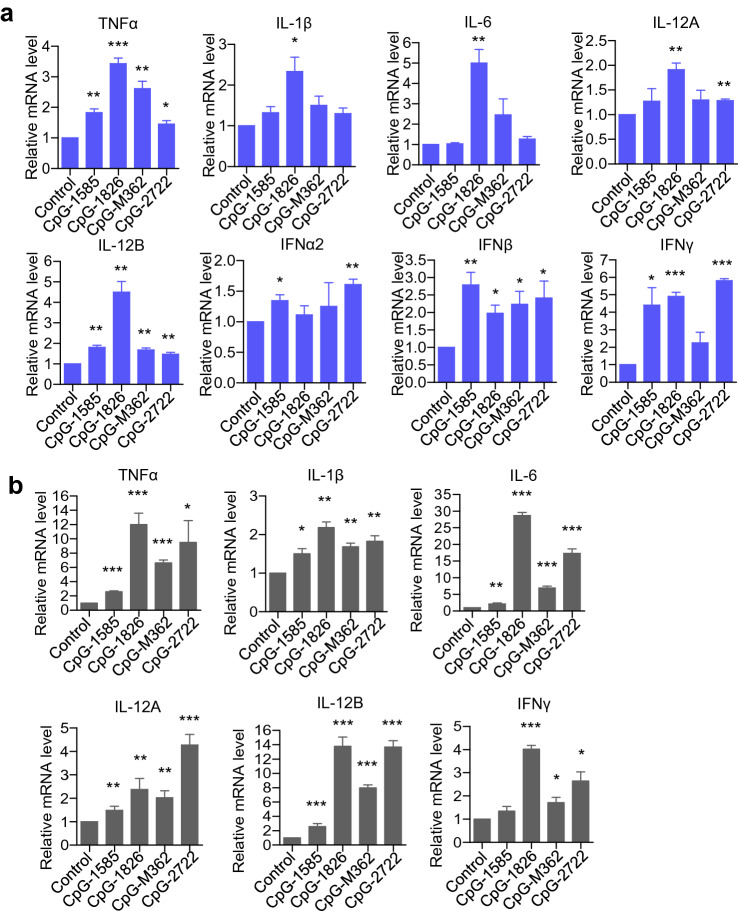
Cytokine-inducing effect of CpG-2722 in mouse cells. **a** Mouse bone marrow-derived macrophages (BMDMs) and **b** mouse splenocytes were treated with 0.5 μM of different CpG-ODNs as indicated for 4 h. Relative mRNA level of cytokines was analyzed by RT-qPCR. Expression level of *β*-actin was used as the loading control. Data represent mean ± SEM (*n* = 3 independent experiments). Asterisk *, **, and *** represent statistically significant difference *p* < 0.05, *p* < 0.01, and < 0.001, respectively, compared to the control

The immunostimulatory activities of CpG-2722 in mouse cells compared to other type of CpG-ODNs were further investigated. In this study, a type A CpG-1585 and type B CpG-1826 with nucleotide sequence designed for the activation of mouse cells were used to replace the CpG-2216 and CpG-2006 used in the studies with human cells (Table 1). Mouse BMDMs were treated with these CpG-ODNs and induction of different gene expressions was analyzed with RT-qPCR. In these cells, the CpG-1826 had better activities in inducing the expression of inflammatory cytokines including TNF-*α*, IL-1*β*, IL-6, and IL-12B than other CpG-ODNs. In contrast, the CpG-2722 had similar activities as this CpG-1826 in inducing the expression of IL-12A and IFN-*γ*. Further, CpG-2722 activated the expression of IFN*α*2 and IFN-*β* as a type A CpG-ODN (Fig. 2a). In addition to these, isolated mouse splenocytes were stimulated with these CpG-ODNs, and gene expression of different cytokines was analyzed. In general, CpG-2722 and CpG-1826, the two type B CpG-ODNs had better activities to induce the expression of these inflammatory cytokines including IL-12A, IL-12B, and IFN-γ compared to the type A and type C CpG-ODNs tested (Fig. 2b).

### Antitumor activity of CpG-2722

**Fig. 3:**
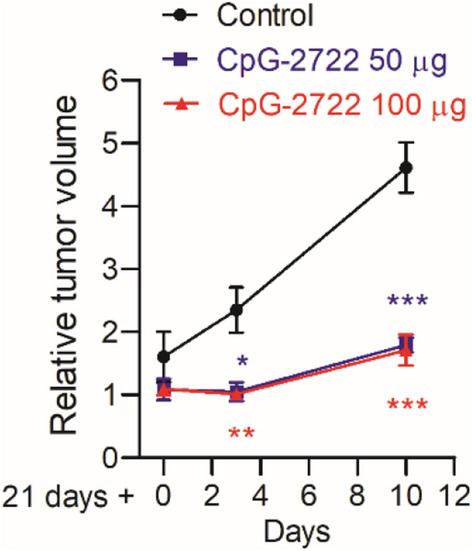
Suppressing effect of CpG-2722 on the growth of head and neck squamous cell carcinoma. C57BL/6 J mice were orthotopically injected with 2 × 10^6^ NHRI-HN1 cells to establish HNSCC. Twenty-one days later, when the tumors reached 250–550 mm^3^, the mice were intratumorally injected with control vehicle, 50 μg, or 100 μg CpG-2722 twice/week. Tumor size was measured at the 21st, 24th, and 31st days (each group contains three mice and three tumors). Data represent mean ± SEM. Asterisk *, **, and *** represent statistically significant difference *p* < 0.05, *p* < 0.01, and *p* < 0.001, respectively, compared to the control

CpG-2722 is capable of inducing the expression of different inflammatory cytokines, including IL-12 and IFN-*γ*, as type B CpG-ODNs and inducing type I interferons as type A CpG-ODNs in both human and mouse cells (Figs. 1 and 2). These cytokines play a critical role in boosting immune responses for the eradication of cancer cells [29, 30]. Therefore, we investigated the antitumor activity of this CpG-ODN. A head and neck cancer cell line, NHRI-HN1, established from C56BL/6 J-derived oral squamous cell carcinoma cells was used for studying the cancer immunobiology of head and neck cancers [26]. These syngeneic NHRI-HN1 cells (2 × 10^6^ cell/mouse) were intramucosally injected into the mice through a side of buccal region to develop orthotopic tumors. Twenty-one days later, when the size of the tumors reached 250–550 mm^3^, and these mice were intratumorally injected with 50 μg or 100 μg of CpG-2722 three times every three days and tumor growth was monitored. The results showed that CpG-2722 effective inhibited tumor growth at both doses (Fig. 3). Therefore, the dose of 50 μg CpG-2722 per mouse was used in the following studies.

### Cooperative effect of combining CpG-2722 and anti-PD-1 on the suppression of head and neck tumor growth

**Fig. 4:**
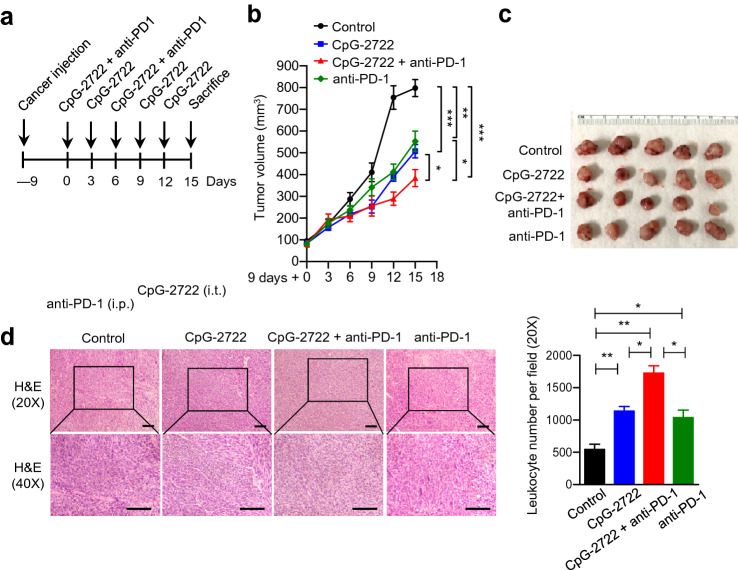
CpG-2722 augments the suppressing effect of immune checkpoint inhibitor on the growth of head and neck squamous cell carcinomas. **a** C57BL/6 J mice were orthotopically injected with 2 × 10^6^ NHRI-HN1 cells to establish HNSCC. Nine days later, when the tumors reached to approx. 100 mm^3^, the mice were intratumorally injected with the control vehicle or 50 μg CpG-2722 every three days in combination with or without the intraperitoneal injection of 10 μg anti-PD-1 antibody once per week for two weeks as illustrated. **b** Tumor sizes were measured every three days (each group contains five mice and five tumors). **c** Endpoint of the tumor growths present as indicated. **d** Tumor samples were visualized by H&E staining for leukocyte infiltrations (left upper panel 20 X, left lower panel 40X). Scale bar represents 100 μm. Leukocyte infiltrations in 20X magnification areas were counted with ImageJ software (Right panel). Data represent mean ± SEM. Asterisk *, **, and *** represent the statistically significant difference *p* < 0.05, *p* < 0.01, and *p* < 0.001, respectively, compared to the control

We further investigated the effect of combining CpG-2722 and anti-PD-1 on the suppression of tumor growth with the NHRI-HN1 syngeneic orthotopic cancer animal model. Two sets of studies were performed. In the first set of experiments, tumors were grown for 9 days to approximately 100 mm^3^, the mice were then continuously intratumorally injected with CpG-2722 every 3 days and intraperitoneally injected with anti-PD-1 at day 0 and day 6 after the injection of CpG-2722 (Fig. 4a). These mice were monitored for the tumor growths (Fig. 4b), euthanized on day 15 after the CpG-2722 and anti-PD-1 treatment, and the tumors were taken for analysis of their sizes (Fig. 4c). Administration of CpG-2722 and anti-PD-1 alone suppressed the tumor growth. Combination of CpG-2722 and anti-PD-1 showed a more effective suppression of the tumor growth than the administration with these two agents alone (Fig. 4b, c). Further, the tumor tissues were hematoxylin and eosin (H&E) stained and number of leukocytes was examined. The result revealed that both CpG-2722 and anti-PD-1 treatments increased the leukocyte infiltration in the tumors, and the combination of CpG-2722 and anti-PD-1 further increased the infiltration (Fig. 4d). In the second set of studies, the experiments performed were similar to those in the first set, except that the frequency of CpG-2722 injection was changed from every three days to every four days, and the route and schedule for administrating anti-PD-1 were substituted by an intravenous injection on days 8 and 16 following the administration of CpG-2722. The results also revealed a cooperative effect of CpG-2722 and anti-PD-1 on the suppression of head and neck tumor growth (Supplementary Fig. 1a–c). Moreover, similar to that in the first set of studies, there was a correlation between the extent of leukocyte accumulation in the tumors and the therapeutic effects of CpG-2722 and/or anti-PD-1 treatments on the suppression of tumor growth (Supplementary Fig. 1d). This suggested activation of inflammatory responses in tumors by the treatments.

### Activation of cytokine expressions in tumor by CpG-2722

**Fig. 5:**
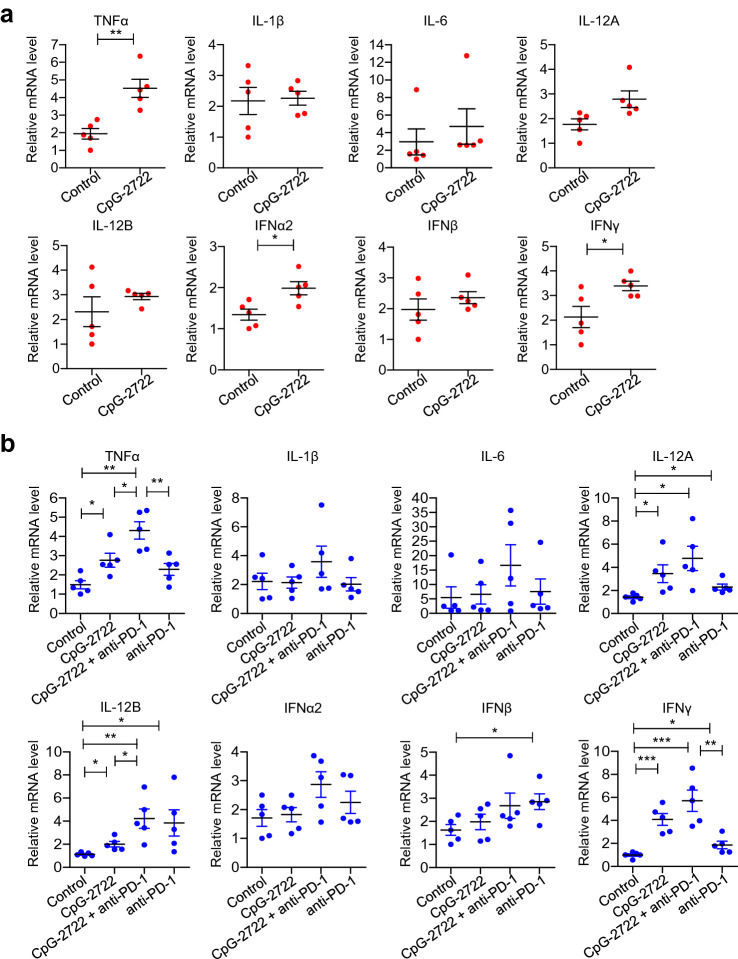
CpG-2722 alone and in combination with immune checkpoint inhibitor augments cytokine genes expression in head and neck squamous cell carcinomas. **a** C57BL/6 J mice were orthotopically injected with 2 × 10^6^ NHRI-HN1 cells and HNSCCs were grown to approx.100 mm^3^. These mice were intratumorally injected with the control vehicle or 50 μg CpG-2722 and euthanized on the next day. **b** Tumor-bearing mice treated with CpG-2722 and anti-PD-1 alone or in combination for 15 days in the experiment for Fig. 4 were euthanized for the collection of tumor samples. Total RNAs from the tumors were isolated by Trizol reagent. The mRNA expression level of different cytokines was measured by RT-qPCR. Expression level of β-actin was used as the loading control. Data represent mean ± SEM. Asterisk *, **, and *** represent statistically significant difference *p* < 0.05, *p* < 0.01, and *p* < 0.001, respectively, compared to the control or as indicated

The mechanism by which the antitumor effect was increased by the combination of CpG-2722 and anti-PD-1 was further investigated. To study cytokine expressions and their kinetics in the CpG-2722-treated tumors, mice were injected with the NHRI-HN1 cells to establish the head and neck cancers. When the tumors reached to approximately 100 mm^3^, the mice were intratumorally injected with CpG-2722 and euthanized 24 h later. Analysis for cytokine expression profiles in the tumors by RT-qPCR revealed that the expression of TNF-*α*, IFN*α*2, and IFN-*γ* genes was induced at the second day after intratumoral injection of the CpG-2722, while the induction of other cytokines was not significant (Fig. 5a). Further, the tumors from the control, CpG-2722, anti-PD-1, and CpG-2722 plus anti-PD-1 15 days continuously treated mice in the experiment of Fig. 4 were analyzed with RT-qPCR for the cytokine expression profiles in the tumors. Gene expression of TNF-*α*, IL-12A, IL-12B, and IFN-*γ* was significantly increased in the tumors derived from the CpG-2722 and CpG-2722 plus anti-PD-1-treated mice (Fig. 5b). The capability of CpG-2722 to induce the expression of these cytokines in tumors is consistent with its ability to induce the expression of these cytokines in immune cells (Figs. 1 and 2). In addition, the profiles of cytokine inductions in tumors on the second day after CpG-2722 injection and at 15 days after the continuous injection of CpG-2722 (Fig. 5) revealed that multiple injections of CpG-ODN are required to achieve an more effective induction of cytokines in the tumors.

### Induction of immune cells accumulation in the tumor microenvironment by CpG-2722

**Fig. 6:**
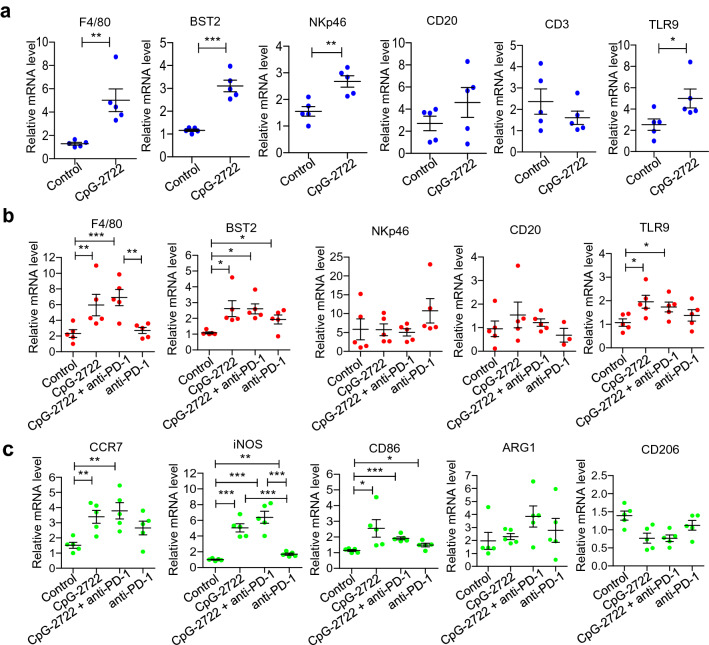
CpG-2722 alone and in combination with immune checkpoint inhibitor increases the accumulation of immune cells in head and neck squamous cell carcinomas. **a** C57BL/6 J mice were orthotopically injected with 2 × 10^6^ NHRI-HN1 cells and HNSCCs were grown to approx.100 mm^3^. These mice were intratumorally injected with the control vehicle or 50 μg CpG-2722 and euthanized on the next day. **b, c** Tumor-bearing mice treated with CpG-2722 and anti-PD-1 alone or in combination for 15 days in the experiment for Fig. 4 were and euthanized for the collection of tumor samples. Total RNAs from the tumors were isolated by Trizol reagent. Expression of markers for different types of immune cells (**a**, **b**), and macrophages (**c**) was analyzed by RT-qPCR. Expression level of β-actin was used as loading control. Data represent mean ± SEM. Asterisk *, **, and *** represent the statistically significant difference *p* < 0.05, *p* < 0.01, and *p* < 0.001, respectively, compare to the control or as indicated

**Fig. 7:**
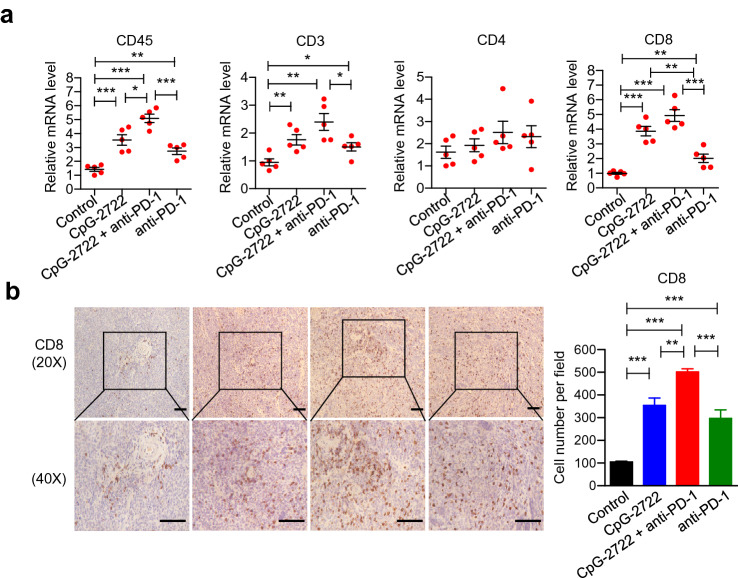
CpG-2722 alone and in combination with immune checkpoint inhibitor increases the accumulation of CD8 positive *T* cells in head and neck squamous cell carcinomas. **a** Tumor-bearing mice treated with CpG-2722 and anti-PD-1 alone or in combination for 15 days in the experiment for Fig. 4 were and euthanized for the collection of tumor samples. Total RNAs from the tumors were isolated by Trizol reagent. Expression of markers for different types of *T* cells was analyzed by RT-qPCR. Expression level of *β*-actin was used as loading control. **b** Immunohistochemistry staining was performed to determine CD8 positive cytotoxic *T*-cell infiltrations (upper panel 20X and bottom panel 40X). Scale bar represents 100 μm. CD8 positive cells were quantified by using ImageJ software at 20X magnification filed (Right panel). Data represent mean ± SEM. Asterisk *, **, and *** represent the statistically significant difference *p* < 0.05, *p* < 0.01, and *p* < 0.001, respectively, compared to the control

The accumulation of different types of immune cells in the tumors was further investigated by RT-qPCR analysis of their cell marker in parallel with the study of the cytokine expressions in the tumors shown in Fig. 5. The results revealed that the expressions of F4/80, BST2, NKp46, and TLR9 but not CD3 and CD20 were induced in the tumors 24 h after the intratumoral injection of CpG-2722 (Fig. 6a). The F4/80, BST2, NKp46, CD3, and CD20 are markers for macrophage, pDCs, NK cells, *T* cells, and *B* cells, respectively. TLR9 is expressed in different immune cells, including dendritic cells, macrophages, natural killer cells, and other antigen-presenting cells. It is more abundant in pDCs and can, therefore, also be used as a marker for pDCs. These results suggested that innate immune cells and the major TLR9 expression cells were accumulated in tumors at the early stage of CpG-2722 stimulation. In contrast, the expressions of F4/80, BST2, and TLR9 but not NKp46 and CD20, were increased in tumors from the 15 days CpG-2722 and CpG-2722 plus anti-PD-1 continuously treated mice (Fig. 6b). Macrophages are a large population of leukocytes in the tumor microenvironment. These tumor-associated macrophages can usually be polarized into two subsets, including inflammatory M1 macrophages, and anti-inflammatory M2 macrophages [31]. CCR7, iNOS, and CD86 are markers for M1 macrophages, and ARG1 and CD206 for M2 macrophages [32, 33]. Analysis of the expression of these markers revealed that the 15 days of continuous treatment with CpG-2722 and anti-PD-1 alone and in combination enhanced the accumulation of M1 macrophages but not M2 macrophages in tumors (Fig. 6c). Accumulation of different *T*-cell subsets following treatments was also investigated. The results revealed an increase in CD3 and CD8 positive *T* cells in the tumors from CpG-2722 and CpG-2722 plus anti-PD-1-treated mice (Fig. 7a). CD8 positive *T* cells play a key role in tumor killing. Histochemical analysis of the tumor tissues further confirmed the results of the RT-qPCR analysis by revealing that the CD8-positive stains were increased in tumor tissues from the mice treated with both CpG-2722 and anti-PD-1 alone and in combination (Fig. 7b).

**Fig. 8:**
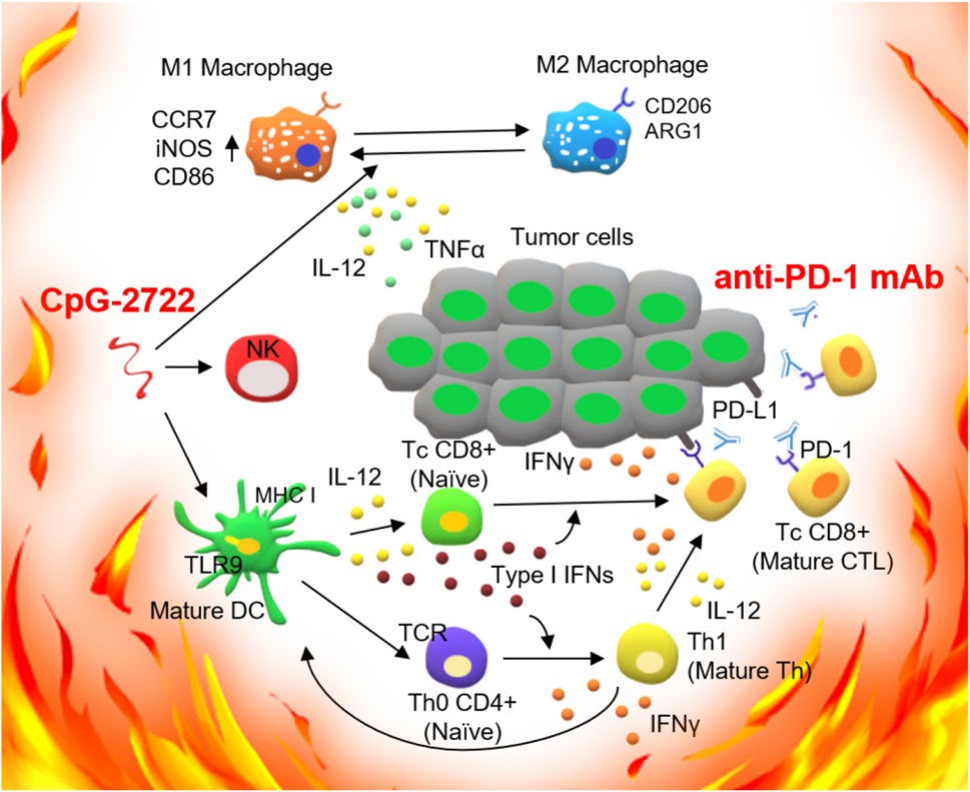
Sharpening up the tumor microenvironment with CpG-2722 increases the efficacy of immune checkpoint blockade. Activation of TLR9 by CpG-2722 triggers immune responses including cytokine production of IL-12 and IFNs and facilitates the accumulation of pDCs, M1 macrophages and CD8 positive cytotoxic *T*-cell in the tumor microenvironment. Immune checkpoint blockade by anti-PD-1 antibody releases the brake of the *T*-cell activation. These events resulted from the combination of CpG-2722 and anti-PD-1, leading to a more effective tumor killing

Macrophages, particularly the M1 macrophages, are the major source of inflammatory cytokines including TNF-*α*, IL-12, and IFN-*γ*. In addition to generating inflammatory cytokines, pDCs are capable of producing IFN-*α* and IFN-*β*. In contrast, NK cells and *T* cells produce cytokines including TNF-*α* and IFN-*γ*; thus, the accumulation of these immune cells in the tumor microenvironment further contributed to the increased levels of cytokines following the CpG-2722 stimulation. In summary, as illustrated in Fig. 8, these results indicate that CpG-2722 is capable of sharpening up the tumor microenvironment by inducing various inflammatory cytokines including IL-12, IFN-*γ*, and type I IFNs, and increasing the accumulation of pDCs, inflammatory M1 macrophages and CD8 positive *T* cells. These immune responses in the tumor microenvironment prime the effector *T* cells for anti-PD-1 to release their brake for tumor killing.

## Discussion

Head and neck cancers comprise a group of malignancies arising from the oral cavity, oropharynx, hypopharynx, larynx and lips, paranasal sinuses, nasopharynx, and nasal cavity. Squamous cell carcinoma (SCC) constitutes the majority (> 90%) of histopathological types of head and neck cancers [34–36]. Immune checkpoint blockade with anti-PD-1 antibodies has been approved by US FDA for the treatment of recurrent and metastatic HNSCC. Nevertheless, the majority of patients do not respond to the therapy, underscoring the need for a strategy to alleviate the resistance of HNSCC to immunotherapy [24, 25]. Previously, only few syngeneic animal models such as the MOC1/2 and TC-1 were available for the study of head and neck cancers. The MOC1/2 cell lines were derived from gene-deficient mice, and the TC-1 cell line was derived from primary lung cells by immortalization and retroviral transduction with HPV16 E6/E740 [37, 38]. Recently, a 4NQO-induced murine oral squamous cell (4MOSC) line was developed, and we developed a stemness-enriched murine HNSCC cell line, NHRI-HN1 for generating syngeneic orthotopic head and neck cancer animal models with C57BL/6 J mice [26, 39]. The NHRI-HN1 cells were demonstrated to have similar gene expression and signaling pathway modulation as human oral squamous cell carcinoma tissues; therefore, the established cancer animal model is reliable for the study of human HNSCC [26]. In this study, we used this newly developed model to study the antitumor effect of combining CpG-2722 and anti-PD-1 on suppressing HNSCC growth.

The CpG-2722 used in this study is a B type CpG-ODN containing 19 nucleotide bases, two copies of GTCGTT-hexamer motifs, and four thymidines in between these two hexamer motifs (Table 1) [27]. CpG-ODN with GTCGTT-hexamer motif often contains species-specific activity to human cells [14–16]. However, in addition to the grouper and human cells, the CpG-2722 also contains immunostimulatory activity to mouse cells. Thus, the CpG-2722 is universal for different species, and research result of this CpG-ODN obtained from cancer animal model is more likely to be replicated in humans. In this study, we further characterized this CpG-ODN and compared its immunostimulatory activity with the CpG-ODNs of different types. CpG-2722 has a good activity on the induction of inflammatory cytokines, particularly the IL-12 genes and IFN-*γ* gene in immune cells, as a type B CpG-ODN. In addition, it also induces the expression of type I IFNs like a type A CpG-ODN. This cytokine-inducing profile is not only seen in immune cells, but it also observed in the tumors of the HNSCC animal models.

The structural basis for the cytokines-inducing profile of CpG-2722 is not clear. Nevertheless, it is known that CpG-ODNs with different structures have different cytokine-inducing profiles. The distinct abilities of type A and type B CpG-ODNs in the induction of type I IFNs are resulted from their higher-order structures. Type A CpG-ODNs are capable of forming multimeric aggregates, whereas type B CpG-ODNs are monomeric and do not have such a feature [40]. A model of spatiotemporal TLR9 activation has been suggested to explain the differential immunostimulatory activities of different CpG-ODNs in dendritic cells. Type A CpG-ODNs activate TLR9 in early endosomes to trigger IRF7 activation for the induction of large amounts of type I IFNs. Type B CpG-ODN is quickly transported to late endosomal/lysosomal compartments for TLR9 activation in order to activate NF-κB and the production of inflammatory cytokines. In contrast, class C CpG-ODNs have the capability to be retained in these endosomal compartments to activate the production of IFNs and inflammatory cytokines [41, 42]. In line with these, encapsulation of class B CpG-ODNs into particles allows their retention in early endosomes for the induction of higher levels of type I IFNs [43]. The CpG-2722 contains activities to induce the production of inflammatory cytokines and IFNs; whether it contains some structural characteristic of the type A CpG-ODNs are still to be investigated.

CpG-2722 had a good activity in inducing IL-12 and IFN-*γ* in different cell types. A heterodimeric form of IL-12 referred to as p70 is composed by the IL-12A (p35) and IL-12B (p40). The promoter of IL-12A contains the binding sites for transcription factors such as NF-κB, c-Rel, and IRF-1. The promoter of IL-12B contains NF-κB, PU.1, IRF-1, IRF-8, NFAT, and AP-1 binding sites. Thus, the production of IL-12 is regulated by multiple signal pathways, leading to the activation of different transcription factors for these binding sites [44–46]. The characteristics of CpG-2722 to regulate multiple pathways including NF-κB and IRF for inductions of inflammatory cytokines and type I IFNs could play some role for its superior activity in regulating the expression of the IL-12 genes. Most of the IL-12-induced antitumor effects include generation of T helper type 1 (Th1) and cytotoxic *T*-cell responses, which are mediated by NK- and *T*-cell-generated inflammatory responses [29, 30]. IL-12 activates the production of IFN-*γ* from these cell types. IFN-*γ* in turn regulates the differentiation and activation of NK cells and *T* cells. In addition, similar to TLR agonists such as CpG-ODN, the IFN-*γ* is effective in inducing the polarization of macrophages into M1 phenotypes. The M1 macrophages are inflammatory, while the M2 macrophages are immunosuppressive cells. These two types of macrophages are convertible. Thus, the increased M1 macrophages in the tumor microenvironment are favorable for generating antitumor responses [31].

The functional mechanisms for the antitumor activities of anti-PD-1 and CpG-ODN have been well investigated. Interaction between PD-1 and its ligands initiates a signal transduction to inhibit TCR activation. In this pathway, PD-1 activates SHP2 tyrosine phosphatase, leading to the dephosphorylation of signaling molecules downstream to TCR and inhibition of TCR activation-mediated cell survival, cell proliferation, and cytokines’ production. Anti-PD-1 blocks this inhibitory signaling, leading to an increased killing activity of CD8* T* cells in the tumors [17, 18]. In contrast, the antitumor activity of CpG-ODN is mainly mediated by inducing the production of inflammatory cytokines from immune cells, which prime the antitumor responses resulting from the activation of immune cells. Of these induced cytokines, IL-12 and IFNs have been shown to contribute to the CpG-ODN-induced antitumor effect [5–7, 47, 48]. CpG-ODN monotherapy often showed good activities in inducing tumor regression in a cancer animal model. In addition, the injection of CpG-ODN into the tumor exerted better antitumor activity than the administration of the CpG-ODN at distant sites such as via intraperitoneal injection or intravenous injection [49, 50]. Based on the positive results of preclinical studies, CpG-ODNs have been investigated in clinical trials as therapeutic antitumor agents [7–9]. However, no CpG-ODN has been approved for cancer treatment so far, suggesting that CpG-ODN alone may not be sufficient for boosting an efficient antitumor immune response in humans. In contrast, although cancer therapy with anti-PD-1 has been approved, only a small portion of patients benefited from this therapy.

The immune system employs coordinated innate immunity and adaptive immunity to elicit an antitumor immune response. The resistance of patients to immunotherapy may result from deficiencies in various aspects of the antitumor response, resulting from an immune-suppressive tumor microenvironment. In this study, we showed the cooperative effect of CpG-2722 and anti-PD-1 on the suppression of HNSCC growth. HNSCC is characterized by an immune-suppressive tumor microenvironment [24, 25]. The function of CpG-2722 to activate various cytokines including IL-12, IFN-*γ*, and type I IFNs leading to the accumulation and activation of immune cells including pDCs, M1 macrophages, and CD8 positive *T* cells in the tumor microenvironment, thus sharpening up the microenvironment into a “hot” one favorable for *T*-cell-mediated tumor killing. Thus, CpG-2722 could be a candidate of combinational therapy with immune checkpoint inhibitors for tumors with an immune suppressive microenvironment.

### Supplementary Information

Below is the link to the electronic supplementary material.

Supplementary file1 (PDF 460 KB)

## Abbreviations

APC: Antigen-presenting cell
BMDM: Bone marrow-derived macrophage
CpG-ODN: CpG-oligodeoxynucleotide
CpG-motif: CpG-dideoxynucleotides containing hexamer motif
CTLA-4: Cytotoxic T-lymphocyte-associated protein 4
DC: Dendritic cell
ELISA: Enzyme-linked immunosorbent assay
FBS: Fetal bovine serum
HNSCC: Head and neck squamous cell carcinoma
IFN-*β*: Interferon-*β*
IFN-*γ*: Interferon-*γ*
IL-6: Interleukin 6
IRF: Interferon regulatory factors
NF-κB: Nuclear factor kappa-light-chain-enhancer of activated B cells
NK: Natural killer cell
PBS: Phosphate buffer saline
PCR: Polymerase chain reaction
PD-1: Programmed cell death protein 1
RT-qPCR: Reverse transcription-quantitative PCR
Th1: T helper 1
TLR: Toll-like receptor
TNF-*α*: Tumor necrosis factor-*α*
