# Supplementary material for: Sharpening up tumor microenvironment to enhance the efficacy of immune checkpoint blockade on head and neck cancer using a CpG-oligodeoxynucleotide
Source: Cancer Immunol Immunother. 2021 Sep 28;71(5):1115–28. doi: 10.1007/s00262-021-03062-8 (PMC9016021; doi:10.1007/s00262-021-03062-8)
Supplement: Supplementary file 1 — Supplementary file1 (PDF 460 KB) [file 262_2021_3062_MOESM1_ESM.pdf]

Supplementary Material

Sharpening up tumor microenvironment to enhance the efficacy of immune checkpoint blockade on head and neck cancer using a CpG-oligodeoxynucleotide

Jen-Chih Tseng[1] · Jing-Xing Yang[1] · Yi-Ling Liu[1] · Yu-Wen Su[1] · Alan Yueh-Luen Lee[2] · Ya-Wen Chen[2] · Ko-Jiunn Liu[2] · Yunping Luo[3] · Yi-Ren Hong[4] · Tsung-Hsien Chuang[1, 5]

[1]Immunology Research Center, National Health Research Institutes, Zhunan, Miaoli 35053, Taiwan.
[2]National Institute of Cancer Research, National Health Research Institutes, Zhunan, Miaoli 35053, Taiwan.
[3]Department of Immunology, Institute of Basic Medical Sciences, Chinese Academy of Medical Sciences; School of Basic Medicine, Peking Union Medical College, Beijing, 100005, China.
[4]Graduate Institute of Medicine, College of Medicine, Kaohsiung Medical University, Kaohsiung 80708, Taiwan.
[5]Program in Environmental and Occupational Medicine, Kaohsiung Medical University, Kaohsiung 80708, Taiwan.

Supplementary table 1 PCR primers for human genes

β-Actin For	CTCTTCCAGCCTTCCTTCCT
β-Actin Rev	TGTTGGCGTACAGGTCTTTG
GAPDH For	GAAGGTGAAGGTCGGAGTCA
GAPDH Rev	AATGAAGGGGTCATTGATGG
IL-1β For	GGACAAGCTGAGGAAGATGC
IL-1β Rev	TCGTTATCCCATGTGTCGAA
IL-6 For	AGACAGCCACTCACCTCTTCAG
IL-6 Rev	TTCTGCCAGTGCCTCTTTGCTG
IL-12A For	TGCCTTCACCACTCCCAAAACC
IL-12A Rev	CAATCTCTTCAGAAGTGCAAGGG
IL-12B For	GACATTCTGCGTTCAGGTCCAG
IL-12B Rev	CATTTTTGCGGCAGATGACCGTG
TNFα For	AACCTCCTCTCTGCCATCAA
TNFα Rev	GGAAGACCCCTCCCAGATAG
IFNα2 For	TGGGCTGTGATCTGCCTCAAAC
IFNα2 Rev	CAGCCTTTTGGAACTGGTTGCC
IFNβ For	TGGGAGGATTCTGCATTACC
IFNβ Rev	CAGCATCTGCTGGTTGAAGA
IFNγ For	TTCAGCTCTGCATCGTTTTG
IFNγ Rev	TCTTTTGGATGCTCTGGTCA

Supplementary table 2 PCR Primers for mouse genes

Gene	Sequence
β-Actin For	GCTACAGCTTCACCACCACA
β-Actin Rev	AAGGAAGGCTGGAAAAGAGC
GAPDH For	AGAACATCATCCCTGCATCC
GAPDH Rev	CACATTGGGGGTAGGAACAC
IL-1β For	AGAGCTTCAGGCAGGCAGTA
IL-1β Rev	AGGTGCTCATGTCCTCATCC
IL-6 For	CCGGAGAGGAGACTTCACAG
IL-6 Rev	TTTCCACGATTTCCCAGAGA
IL-12A For	ACGGCCAGAGAAAAACTGAA
IL-12A Rev	CTACCAAGGCACAGGGTCAT
IL-12B For	CACGCCTGAAGAAGATGACA
IL-12B Rev	AGTCCCTTTGGTCCAGTGTG
TNFα For	ACGGCATGGATCTCAAAGAC
TNFα Rev	GTGGGTGAGGAGCACGTAG
IFNα2 For	ATCCAGAAGGCTCAAGCCATCC
IFNα2 Rev	GGAGGGTTGTATTCCAAGCAGC
IFNβ For	GCCTTTGCCATCCAAGAGATGC
IFNβ Rev	ACACTGTCTGCTGGTGGAGTTC
IFNγ For	CAGCAACAGCAAGGCGAAAAAGG
IFNγ Rev	TTTCCGCTTCCTGAGGCTGGAT
CD3 For	CTGCTACACACCAGCCTCAA
CD3 Rev	GCCTTGGCCTTCCTATTCTT
CD4 For	CTGATGTGGAAGGCAGAGAAG
CD4 Rev	GAGACCTGGGGTATCTTGAGG
CD8 For	TATGGCTTCATCCCACAACA
CD8 Rev	GACTGGCACGACAGAACTGA
CD45 For	TCAGCAAGTTTCCCATCAAAG
CD45 Rev	CCCTTGTGCAGCAATGTATTT
CD20 For	GTCTTCGCACCCATCTGTTT
CD20 Rev	AATGGCAGCAAAGAGGCTTA
CD86 For	TCAGTGATCGCCAACTTCAG
CD86 Rev	TTAGGTTTCGGGTGACCTTG
F4/80 For	CTCTGTGGTCCCACCTTCAT
F4/80 Rev	GATGGCCAAGGATCTGAAAA
CCR7 For	AACGGGCTGGTGATACTGAC
CCR7 Rev	TAGGCCCAGAAGGGAAGAAT
CD206 For	TTTGAGTGGAGTGATGGAACC
CD206 Rev	ACAGCATGGCTTTGTGATACC

iNOS For	CACCTTGGAGTTCACCCAGT
iNOS Rev	ACCACTCGTACTTGGGATGC
ARG1 For	ACAGGGCTCCTTTCAGGACTA
ARG1 Rev	AGTCAGTCCCTGGCTTATGGT
BST2 For	CAATCTACTTCGCCGTCACA
BST2 Rev	TCTTCTCCAGGGACTCCTGA
NKp46 For	ACTGTGCCTTGGGCTATGTC
NKp46 Rev	CTCGATGGCTTTGGTCTCTC
TLR9 For	TTCTCTTCATGGACGGGAAC
TLR9 Rev	GGCACCTTTGTGAGGTTGTT

Supplementary figure 1

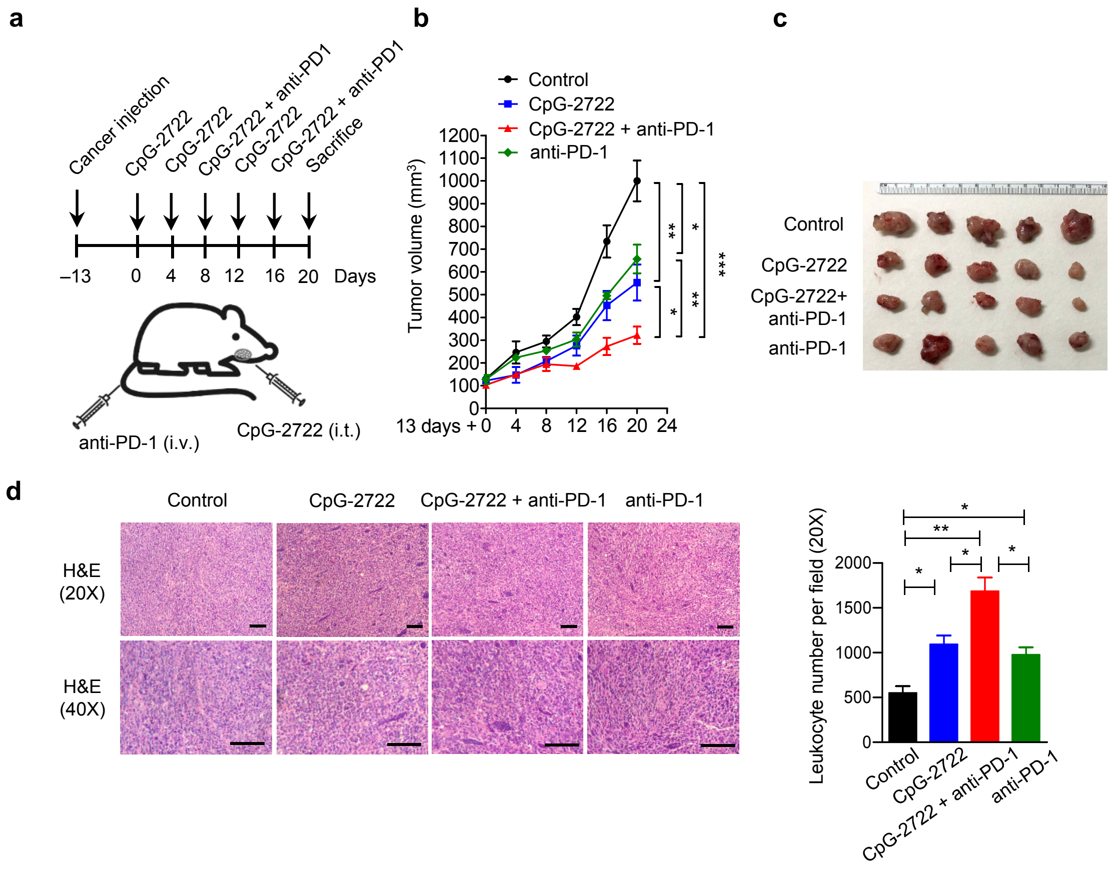

Supplementary Fig. 1 CpG-2722 augments suppressive effect of intravenous injected immune checkpoint inhibitor on growth of head and neck squamous cell carcinomas. **a** C57BL/6J mice were orthotopically injected with 2×10^6 NHRI-HN1 cells to grow HNSCC. Thirteen days later, when the tumors reached approx. 100 mm^3, the mice were intratumorally injected with the control vehicle or 50 µg CpG-2722 every four days in combination with or without the intravenous injection of 10 µg anti-PD-1 antibody every eight days as illustrated. **b** Tumor sizes were measured (each group contained five mice and five tumors). **c** Endpoint of the tumor growths are represented as indicated. **d** Tumor samples were visualized by H&E staining (left upper panel). Scale bar represents 100 µm. Leukocyte infiltrations in the 20x magnification areas were counted by using Image J software (Right panel). Data represent mean ± SEM. Asterisk *, **, and *** represent the statistically significant difference $p < 0.05$, $p < 0.01$, and $p < 0.001$, respectively, compared to the control.
